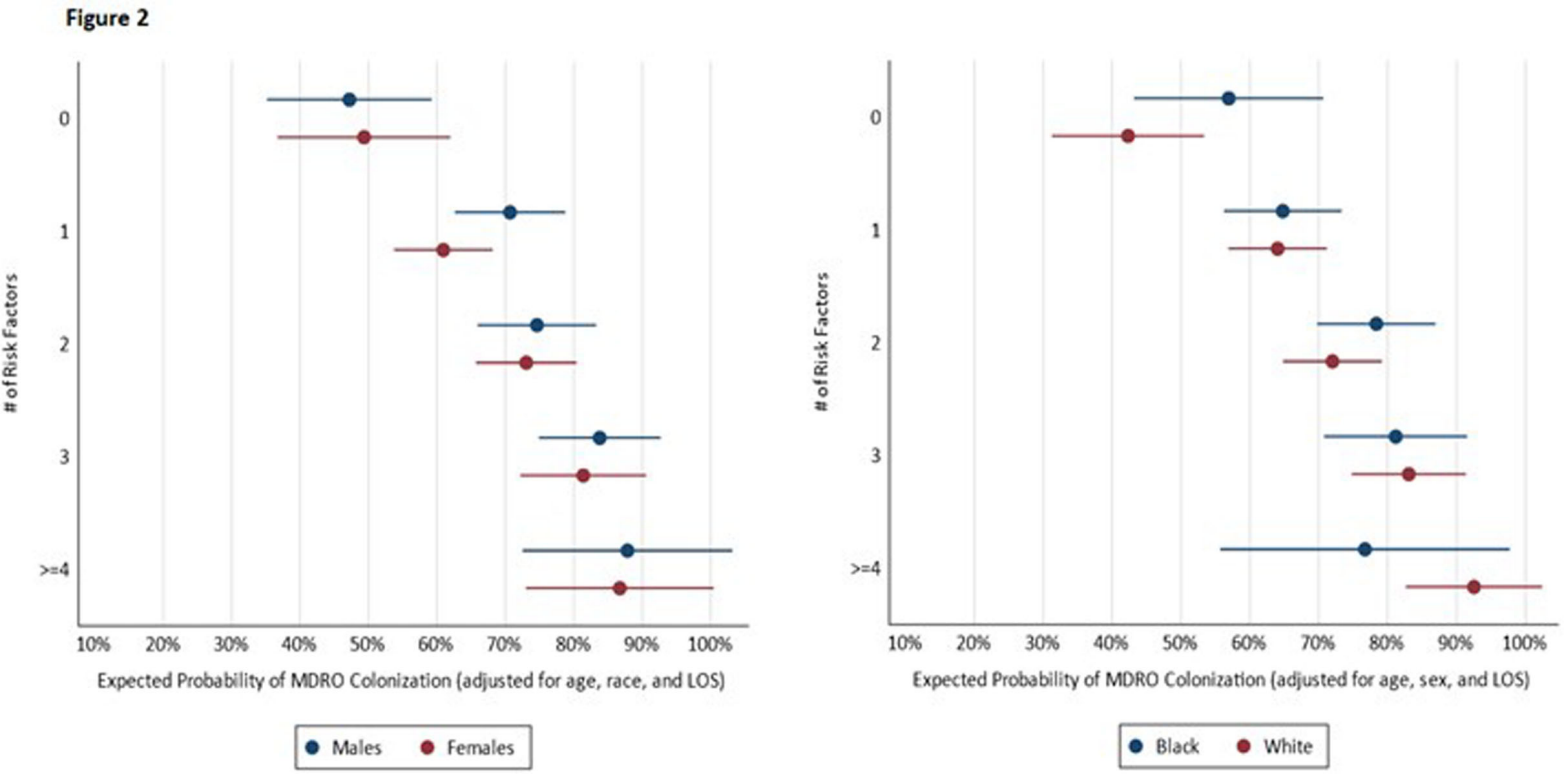# MDRO Colonization Among Nursing Homes Patients: A Risk Classification Tool for Early Identification

**DOI:** 10.1017/ash.2024.275

**Published:** 2024-09-16

**Authors:** Ganga Vijayasiri, Lona Mody, Kristen Panson, Marco Cassone, Andrzej Galecki

**Affiliations:** University of Michigan; University of Michigan and VA Ann Arbor Healthcare System

## Abstract

**Background:** Nursing homes (NHs) have high prevalence of multi-drug resistant organisms (MDROs) with rates exceeding those in hospitals. This study proposes quantifying patients’ risk of MDRO colonization and creating a risk profile for the NH patient populations to assist in reducing MDRO burden in health care facilities. **Methods:** We assessed a risk classification model using data from a prospective cohort study (2018 Pathways study). Patient sample included 783 newly admitted patients followed for up to 180 days and 9,587 samples collected from patients during 2,089 visits. Individual risk factors of MDRO colonization were assessed using unadjusted logistic regression, and patients were risk classified based on number of risk factors. Multivariate regression was performed to obtain odds of MDRO colonization for patient risk groups adjusting for patient age, sex, race, and length of NH stay (LOS). The risk classification tool developed using Pathways data was also tested among a sample of NH residents from Veterans Administration (N=190). **Results:** The patient sample (Pathways data) was 43.2% male, 37.2% Black with a mean age 74 years. 69.3% were colonized with a MDRO during the study. In unadjusted regression, recent antibiotic use (p<.001), open wounds (p<.05), use of urinary catheter or feeding tube (p<.001), functional disability (p<.001), diabetes (p<.01), and preadmission hospital stay over 14 days (p<.001) were associated with colonization, while Charlson comorbidity score, age, sex, and race were not. In adjusted analysis (c-statistic=0.75), a patient’s colonization risk increased with the number of risk factors (Figure 1), with 47.2% expected colonization among patients with none of the risk factors and 86.7% expected colonization among patients with four or more risk factors. The risk classification model had similar performance among male and female patients, and among Black and White patients (Figure 2). Secondary analysis using data obtained from separate, Veterans Administration facilities provided preliminary validation of the risk scoring tool. The model had acceptable fit (c-statistic=0.71). Veterans with four or more risk factors had 87.8% expected probability of MDRO colonization compared with 39.6% colonization among those without any risk factors. Veterans with less than four risk factors also had higher colonization, but these differences were not statistically significant. Conclusions. Despite system-wide efforts to reduce MDRO burden, prevalence of MDRO colonization remains high in NHs. The risk classification tool can assist in early identification of most vulnerable NH patients to direct targeted interventions such as education, enhanced environmental cleaning, and active surveillance.

**Disclosure:** Lona Mody: NIH, VA, CDC, Kahn Foundation; Honoraria: UpToDate; Contracted Research: Nano-Vibronix

**Figure:**
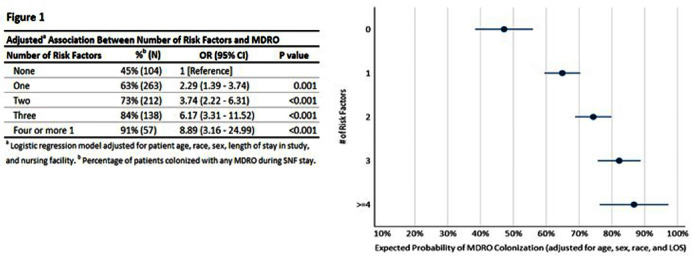

**Figure:**